# Case for diagnosis. Subcutaneous nodules in the plantar region^☆^^☆☆^

**DOI:** 10.1016/j.abd.2020.02.006

**Published:** 2020-06-26

**Authors:** Diego Henrique Morais Silva, Isaura Azevedo Fasciani, Neusa Yuriko Sakai Valente, Bethânia Cabral Cavalli Swiczar

**Affiliations:** Department of Dermatology, Hospital do Servidor Público Estadual de São Paulo, São Paulo, SP, Brazil

**Keywords:** Arthritis, rheumatoid, Granuloma, Rheumatoid nodule

## Abstract

The authors report a case of mobile and painful nodules on the bilateral plantar surface of a female patient referred by the rheumatology service, where she was being followed-up for rheumatoid arthritis. A nodule excision was performed for differential diagnosis and symptom relief; the histopathological analysis was compatible with a rheumatoid nodule. Although rheumatoid nodules are a common manifestation of rheumatoid arthritis, exclusive plantar involvement is seldom described in the literature.

## Case report

**Figure 1:**
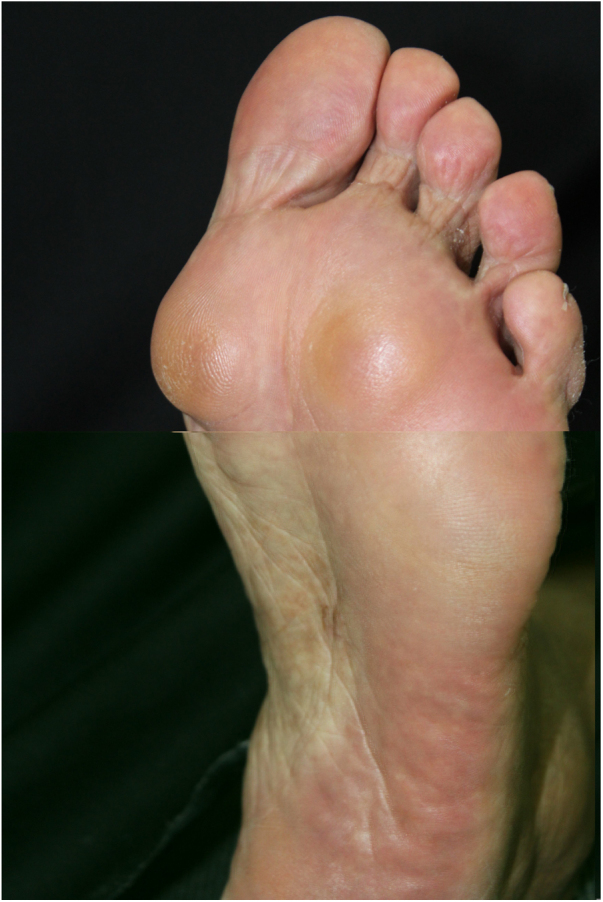
Nodules on the left plantar surface.

**Figure 2:**
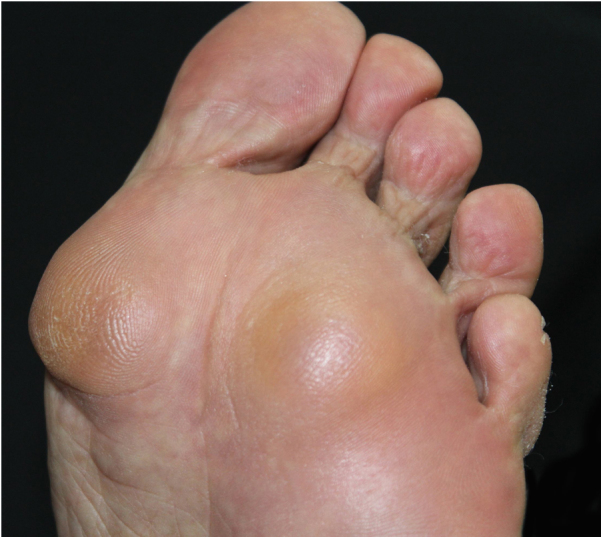
Detail of nodules in pressure areas of the plantar region.

**Figure 3:**
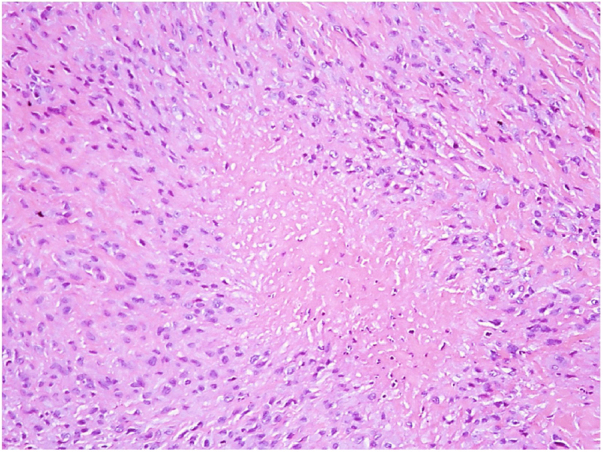
Granuloma with central necrosis surrounded by a palisade of epithelioid cells (Hematoxylin & eosin, ×100).

A 60-year-old caucasian woman was referred to the dermatology outpatient clinic by the rheumatology service, where she was followed-up due to a 20-year history of rheumatoid arthritis. The patient reported a two-year history of painful nodules on the soles of her feet, with slow growth, which hindered walking (Figure 1, Figure 2). The ultrasound report showed nonspecific nodules in the subcutaneous tissue. The appearance of the nodules was accompanied by a progressive worsening of joint symptoms. One of the nodules was excised (Fig. 3).

## What is your diagnosis?

**a)** Fibroma
**b)** Rheumatoid nodule
**c)** Lipoma
**d)** Calcinosis

## Discussion

Rheumatoid arthritis is a systemic inflammatory disease, primarily articular, that affects about 0.8% of the world population.^1^ Among the cutaneous manifestations of rheumatoid arthritis, rheumatoid nodules are the most common, being observed in approximately 30% of these patients. They typically consist of subcutaneous, painless nodules located in extensor and repetitive trauma regions such as the back of the hands, ankles, knees, elbows, and the occipital region.^2^ Rheumatoid nodules are more common in men, especially Caucasians.^1^ Plantar involvement is rare and seldom described in the literature; it is generally observed in the context of multiple lesions in other locations.^3^

There are a variety of differential diagnoses, including subcutaneous granuloma annulare, calcinosis, lipoma, and gouty tophi; however, the history of rheumatoid arthritis, especially in a context of exacerbation of the articular condition, favors the clinical diagnosis of rheumatoid nodule. In doubtful cases, the definitive diagnosis is established by histopathological findings of a granulomatous process characterized by a focus of fibrinoid necrosis surrounded by a palisade of histiocytes.^2^ Most patients have asymptomatic nodules and do not require treatment; however, when these nodules become painful, infected, or ulcerated, treatment is imperative.^1^, ^2^, ^4^ The intralesional application of corticosteroids to reduce the size of the nodules is the treatment of choice in most symptomatic cases. Surgical resection is generally not necessary, except in cases of nerve compression or those with range of joint movement limitation.^1^, ^4^

After excision and confirmation of the diagnosis of rheumatoid nodule, the patient is currently being followed-up by the rheumatology team, with improvement of joint symptoms after the introduction of certolizumab, which inhibits tumor necrosis factor-α (TNF-α). As the joint symptoms were controlled, the patient presented an improvement in symptoms and a reduction in the dimensions of the plantar nodules.

## Financial support

None declared.

## Authors' contributions

Diego Henrique Morais Silva: Conception and planning of the study; elaboration and writing of the manuscript.

Isaura Azevedo Fasciani: Conception and planning of the study; critical review of the literature.

Neusa Yuriko Sakai Valente: Conception and planning of the study; obtaining, analyzing, and interpreting the data; effective participation in research orientation; intellectual participation in propaedeutic and/or therapeutic conduct of studied cases.

Bethânia Cabral Cavalli Swiczar: Effective participation in research orientation; intellectual participation in propaedeutic and/or therapeutic conduct of studied cases.

## Conflicts of interest

None declared.
